# Respiratory Paradoxical Adverse Drug Reactions Associated with Acetylcysteine and Carbocysteine Systemic Use in Paediatric Patients: A National Survey

**DOI:** 10.1371/journal.pone.0022792

**Published:** 2011-07-27

**Authors:** Pauline Mallet, Nadjette Mourdi, Jean-Christophe Dubus, Françoise Bavoux, Marie-José Boyer-Gervoise, Marie-Josèphe Jean-Pastor, Martin Chalumeau

**Affiliations:** 1 Department of pediatrics, Necker-Enfants Malades hospital, Université Paris Descartes, Assistance Publique – Hôpitaux de Paris, Paris, France; 2 Regional Pharmacovigilance Center and Department of Clinical Pharmacology, Cochin Saint Vincent de Paul Hospital, Université Paris Descartes, Assistance Publique – Hôpitaux de Paris, Paris, France; 3 Inserm U953, Saint-Vincent-de-Paul hospital, Paris, France; 4 Unité de Pneumologie et Médecine Infantile, CNRS URMITE 6236, CHU Timone-Enfants, Marseille, France; 5 Regional Pharmacovigilance Center, Salvator Hospital, Assistance Publique – Hôpitaux de Marseille, Marseille, France; Dr. Margarete Fischer-Bosch Institute of Clinical Pharmacology, Germany

## Abstract

**Objective:**

To report pediatric cases of paradoxical respiratory adverse drug reactions (ADRs) after exposure to oral mucolytic drugs (carbocysteine, acetylcysteine) that led to the withdrawal of licenses for these drugs for infants in France and then Italy.

**Design:**

The study followed the recommendations of the European guidelines of pharmacovigilance for medicines used in the paediatric population.

**Setting:**

Cases voluntarily reported by physicians from 1989 to 2008 were identified in the national French pharmacovigilance public database and in drug company databases.

**Patients:**

The definition of paradoxical respiratory ADRs was based on the literature. Exposure to mucolytic drugs was arbitrarily defined as having received mucolytic drugs for at least 2 days (>200 mg) and at least until the day before the first signs of the suspected ADR.

**Results:**

The non-exclusive paradoxical respiratory ADRs reported in 59 paediatric patients (median age 5 months, range 3 weeks to 34 months, 98% younger than 2 years old) were increased bronchorrhea or mucus vomiting (n = 27), worsening of respiratory distress during respiratory tract infection (n = 35), dyspnoea (n = 18), cough aggravation or prolongation (n = 11), and bronchospasm (n = 1). Fifty-one (86%) children required hospitalization or extended hospitalization because of the ADR; one patient died of pulmonary oedema after mucus vomiting.

**Conclusion:**

Parents, physicians, pharmacists, and drug regulatory agencies should know that the benefit risk ratio of mucolytic drugs is at least null and most probably negative in infants according to available evidence.

## Introduction

Cysteine derivatives (carbocysteine, acetylcysteine) are mucolytic drugs that act by breaking disulphide bridges between macromolecules and lead to reduced mucus viscosity in the respiratory tract [1]. These derivatives are widely used for paediatric patients to treat acute respiratory tract infections such as bronchitis, bronchiolitis, or productive cough. They are some of the most prescribed medicines for children, particularly infants in European countries such as The Netherlands [2], Italy [3], Spain [4], France [5]–[7], and Germany [8], and in Brazil [9] and African countries [10].

Several alerts have concerned the safety and benefit risk ratio of cough medicines for children, particularly during recent years. In January 2008, the US Food and Drug Administration recommended not to use over-the-counter medicines for children under 2 years old, particularly medicines for cough and cold, because of serious and potentially life-threatening side effects [11]. In 2010, the French drug agency, and then the Italian drug agency, withdrew the licenses for children younger than 2 years old for carbocysteine and acetylcysteine because their use was associated with paradoxically increased bronchorrhea and acute respiratory distress during respiratory tract infections [12]–[14]. However, published data on these paradoxical adverse drug reactions (ADRs) are limited to short hospital-based studies with a very small number of cases (n = 6) [12] or were simply mentioned in the discussion of a Cochrane database systematic review without description of cases [13].

Carbocysteine and acetylcysteine are still licensed and widely used to treat acute cough in infants in various European and African countries, and millions of children have been exposed to these drugs, despite a lack of evidence of efficacy as shown by the Cochrane database systematic review and strong warnings on safety [12]–[16]. Detailed data on the ADRs that led to the withdrawal of the license in France could help parents (because these drugs are over-the-counter drugs), physicians, pharmacists, and drug regulatory agencies precisely evaluate the benefit risk ratio of these drugs. We aimed to analyze cases of respiratory ADRs during the use of mucolytic drugs in children, particularly infants, voluntarily reported by physicians in France.

## Methods

### General methodology

The study followed the recommendations of the European Guidelines of pharmacovigilance for medicines used in the paediatric population [17], the guidelines for submitting adverse events reports for publication [18], and the French pharmacovigilance practice guidelines [19]. We analyzed all cases of respiratory paradoxical ADRs (as described below) concerning carbocysteine and acetylcysteine voluntarily reported from 1989 to 2008 to the French pharmacovigilance system. In France, physicians and pharmacists must report all potential ADRs to public regional centers of pharmacovigilance or national drug company pharmacovigilance centers using pre-define forms [20]. The forms are completed anonymously and contain data on age, weight, medical history of the patient, drug exposure, and the side effect reported (type, severity, co-treatments received). Cases are then analyzed by pharmacologists; additional data can be collected from the declaring physicians or pharmacists before the file is validated and included anonymously in the national database or the drug company databases.

For the present study, the case identification strategy involved use of the national pharmacovigilance website ARIS g5 (containing cases reported to regional centers of pharmacovigilance) and reports by the industry of any case of ADR involving carbocysteine and acetylcysteine during the national survey conducted after a commitment of the French drug agency [14], with an age limit of 0 to 6 years. We hand-searched results and excluded cases of non-respiratory ADR.

### Cases

Our investigation focused on potential ADRs reported in the literature [13] or described in the warning report [14] of the French drug agency: worsening of a respiratory disease (during bronchitis, bronchiolitis, or pneumonia), increased bronchorrhea during acute cough, mucus vomiting, dyspnea, prolonged cough, and bronchospasm. We included all cases whatever the medical history or chronic underlying disease.

### Exposure definition

In France, until April 2010, 42 formulations of carbocysteine (in syrup or powder) and 15 of acetylcysteine were licensed for use in paediatric patients from 1 month of age. These drugs are to be absorbed by an oral route, and no license exists for the nebulized route of administration. For carbocysteine, the recommended dosage for children from 1 month to 2 years is 20 to 30 mg/kg/day divided into 2 to 3 intakes. For children from 2 to 5 years old, the recommended dosage is 100 mg twice a day and for those older than 5 years, 100 mg 3 times a day. For acetylcysteine, the recommended dosage is 100 mg twice a day for infants from 1 month to 2 years old and 200 mg twice a day for children older than 2 years. The duration of the treatment should not exceed 8 to 10 days without medical advice [21].

For the present study, patients were arbitrarily considered exposed to mucolytic drugs if they had received carbocysteine or acetylcysteine for at least 2 days (>200 mg) and at least until the day before the first signs of the suspected ADR.

### Analysis

We first described the population of patients included in the study, then analyzed the indications of the prescription, the exposure to mucolytic drugs, the potential ADRs reported and their evolution, then co-treatments received.

### Ethics

The Institutional Review Committee (Comité de Protection des Personnes Ile de France III) approved this study. Written informed consent of the patients or their parents was not possible for this kind of study based on anonymous data. The waiver of consent was approved by the Institutional Review Committee (Comité de Protection des Personnes Ile de France III).

## Results

During the study period, 1989 to 2008, 139 cases of ADRs in children (all younger than 6 years) potentially due to mucolytic drug exposure were reported to public regional centers of pharmacovigilance or to drug companies. We excluded 69 reports of non-respiratory ADRs and 11 of respiratory ADRs with a total drug exposure <200 mg. Thus, the study analyzed the data for 59 cases of respiratory ADR in children younger than 6 years after an exposure of >200 mg to mucolytic drugs. Seven cases had been reported to drug companies, the 52 other cases had been reported to 6 regional centers of pharmacovigilance throughout France. Among the 59 cases, 46 cases (78%) were reported in the last 7 years (2002–2008) of the study period. The mean age of children was 8.6 months (median age 5 months, range 3 weeks to 34 months): 6 children (10%) were neonates, 34 (58%) were younger than 6 months, 46 (78%) were younger than 1 year old, and 11 (19%) were between 1 and 2 years old, and one child was older than 2 years. The mean weight was 7.9 kg (median 7 kg, range 3.1–18 kg). One patient had a severe underlying condition (broncho-pulmonary dysplasia); 9 children (15%) had a relevant medical history prior to the current episode that led to mucolytic drug use: bronchiolitis (n = 8, 14%), asthma (n = 1).

At the time of the mucolytic drug use, the non-exclusive symptoms or diagnoses were cough (n = 28, 47%), rhinitis (n = 14, 24%), rhinopharyngitis (n = 8, 14%), bronchiolitis (n = 8, 14%), bronchitis (n = 7, 12%), dyspnoea (n = 6, 10%), bronchorrhea (n = 4, 7%), tracheitis (n = 1), pleuropneumonia (n = 1), and fever (n = 1).

Thirty patients (51%) received carbocysteine and 28 (48%) acetylcysteine, and one child received the 2 drugs simultaneously. No child was exposed to a drug combination such as carbocysteine–promethazine. The mean duration of mucolytic treatment before the beginning of the ADR was 5.9 days (median 4 days; range 2–39 days). All children received oral treatment. Four children (7%) received this treatment without a prescription (i.e., over-the-counter drug). For carbocysteine, the mean dosage was 181 mg per day (median 200 mg; range 100–300 mg), which represented a mean of 23 mg/kg per day (median 23 mg/kg; range 8–54 mg/kg). For acetylcysteine, the mean drug regimen was 182 mg per day (median 200 mg; range 100–400 mg) and 27 mg/kg per day (median 22 mg/kg; range 8–65 mg/kg). Patients younger than 6 months received a mean drug regimen of 28 mg/kg per day as compared with 16 mg/kg per day for older children.

The non-exclusive respiratory ADRs reported were worsening of respiratory distress during bronchiolitis (n = 35, 59%), increased bronchorrhea (n = 19, 32%), dyspnoea (n = 18, 31%), cough aggravation or prolongation (n = 11, 19%), mucus vomiting (n = 8, 14%), pneumonia (n = 3, 5%), acute bronchitis (n = 2, 3%), and bronchospasm (n = 1).

Fifty-one children (86%) required hospitalization or extended hospitalization because of the ADR. Mucolytic drug treatment was withdrawn for all patients. Oxygenotherapy was required for 18 children (31%); 12 (20%) received antibiotics, 9 (15%) corticosteroids for asthma crisis, and 11 (19%) inhaled β2 agonists. Evolution was favorable in all cases except for one patient in whom pleuropneumonia developed and a 1-year-old girl who died of pulmonary oedema that was considered secondary to mucus vomiting according to the reporting physician.

During the same window of exposure as that chosen for mucolytic drugs, 21 children (36%) had received corticosteroids, 21 (36%) antibiotics, 12 (20%) inhaled β2 agonists, 11 (19%) acetaminophen, 5 (8%) ibuprofen, 4 (7%) domperidone, one helicidine, one oxomemazine and guaifenesine, and 3 (5%) antihistamine (ketotifen, mequitazine, chlorphenamine).

## Discussion

This study is the first to analyze cases of respiratory ADRs associated with the systemic use of carbocysteine and acetylcysteine in children that were reported to the pharmacovigilance reporting system. We identified 59 cases of worsening of a respiratory disease increased bronchorrhea during acute cough, dyspnoea, prolonged cough, mucus vomiting, and bronchospasm. These cases were severe: 51 patients (86%) required hospitalization or extended hospitalization, and 1 patient died.

The literature provides a hypothesis to explain the mechanisms by which use of mucolytic drugs during acute respiratory tract infections in infants may be associated with respiratory ADRs [12]–[16]. The anatomical and physiological specificities of the airways of infants – numerous mucus glands, small bronchial diameter, muscular immaturity – predispose this age group to this kind of ADR [22]. During infection, the modifications of the airway (i.e., bronchorrhea, reduced bronchial diameter, inflammation) enhance this predisposition. The increase in bronchial mucus flow induced by mucolytic drugs could exceed the ability for spontaneous drainage in the infant and then induce prolonged cough and worsened respiratory distress during acute airway infection. Interestingly, this kind of respiratory ADR was mentioned early in reviews [23], [24] and pharmacological textbooks [25], [26] but was not clinically documented or declared to the pharmacolovigilance system.

Despite these strong pathophysiological bases, a causal relationship would be difficult to definitively prove in these cases of respiratory paradoxical ADRs associated with the systemic use of carbocysteine and acetylcysteine in pediatric patients because of the potential presence of a protopathic bias. Protopathic bias can occur when the first symptoms of the potential ADR can induce treatment. This bias could be present with respiratory ADRs associated with the systemic use of carbocysteine and acetylcysteine in pediatric patients. The only way to evaluate the paradoxical side effects of drugs and avoid protopathic bias would be theoretically to study them in another unrelated disease [13]. However, this is not possible in the case of carbocysteine because its only indications are respiratory tract infections. For acetylcysteine, data from a multicenter postmarketing safety study including 1905 pediatric patients receiving acetylcysteine for acetaminophen overdose between 1980 and 2005 are consistent with this hypothesis of paradoxical side effects [27]. Indeed, in this study where patients did not receive acetylcysteine to treat respiratory tract infection, the incidence of respiratory ADRs (such as bronchospasm, cough, wheezing, or respiratory distress) was 2.2% [27]. Thus, the level of evidence of the respiratory paradoxical ADRs associated with the systemic use of carbocysteine and acetylcysteine in pediatric patients will probably stay limited to experts' opinion and strong pathophysiological bases.

That young infants are more affected than older ones by respiratory ADRs associated with the systemic use of carbocysteine and acetylcysteine seems to have 2 explanations. First, the pediatric anatomical particularities described previously are more important in the youngest infants. Second, the dosage proposed in the license for acetylcysteine indicates a single dose for children from 1 to 24 months whatever the weight of the child. According to World Health Organization standards, the median weight at 1 month and 2 years is 4.5 and 12.2 kg, respectively [28]. A single dose regimen for children from 1 to 24 months induces a different regimen per weight (44.4 vs. 16.4 mg/kg/day) and exposes the youngest infants to higher doses, as was observed in our study. Thus, a dose-related effect could explain the age distribution of the reported cases of respiratory ADRs.

We could not evaluate the frequency of respiratory ADRs among pediatric patients. Safety data from clinical studies performed by drug companies did not identify these respiratory ADRs [13]. However, the power of the efficacy studies was limited by the small number of patients included (n<152) and the very small number of infants included (n<29) [13]. The power of the unique study designed to evaluate safety was low (n = 20), and this study included few infants [29]. The total number of ADRs we describe could seem low as compared with the huge extent of the exposure of the pediatric population to these drugs. However, the lack of exhaustiveness of a spontaneous reporting system for ADRs is well known. Begaud *et al.* showed only 5% of severe ADRs leading to the hospitalization of patients reported in France [30]. Thus, the exact number of respiratory ADRs that occur in the population could be much higher than that reported and analyzed in this survey. For example, a 2-month prospective study performed in the emergency department of 2 pediatric teaching hospitals in Paris, France, identified 6 cases [12], [13]. The frequency of respiratory ADR was not calculated in the present study and is most probably very low. However, it does not modify the evaluation of the benefit risk ratio of these drugs for which there is a complete lack of evidence of efficacy as shown by the Cochrane database systematic review [13].

In conclusion, carbocysteine and acetylcysteine are still licensed and largely used for children younger than 2 years in many European, South-American and African countries, despite a clearly demonstrated lack of evidence of efficacy and a strong warning on safety as reported in this study and others [12]–[16]. Thus, parents, physicians, pharmacists, and drug regulatory agencies should know that the benefit risk ratio of these drugs is at least null and most probably negative in this age group according to available evidence.
